# Bovine leukemia virus DNA associated with breast cancer in women from South Brazil

**DOI:** 10.1038/s41598-019-39834-7

**Published:** 2019-02-27

**Authors:** Daniela Schwingel, Ana P. Andreolla, Luana M. S. Erpen, Rafael Frandoloso, Luiz C. Kreutz

**Affiliations:** Universidade de Passo Fundo, Laboratório de Microbiologia e Imunologia Avançada, Prédio G3. Campus I, Bairro São José, BR 285, Km 292, 99052-900 Passo Fundo, RS Brazil

## Abstract

Breast cancer is a neoplastic condition with a high morbidity and mortality amongst women worldwide. Recent data linking bovine leukemia virus (BLV) with breast cancer has been contested already. Our study investigated the presence of BLV genome in healthy (n = 72) and cancerous (n = 72) paraffin-embedded samples of breast tissues from women in south Brazil. BLV DNA was found most frequently (30.5%) in breast cancer tissue than in healthy breast (13.9%) (Odds ratio = 2.73; confidence interval = 1.18–6.29; p = 0.027). In contrast, antibodies to BLV were found in a very small percentage of healthy blood donors. There was no association between BLV DNA and other tumor prognostic biological markers such as hormonal receptors, HER2 oncoprotein, proliferation index, metastasis in sentinels lymph nodes, and tumor grade and size. Our findings suggest that BLV should be considered a potential predisposing factor to breast cancer in women.

## Introduction

Globally, more than 1.7 million new cases of breast cancer in women are diagnosed each year; half of them, and approximately 60% of breast cancer-associated deaths occur in women from developing countries^1^. In Brazil, there were 58 thousand new cases of breast cancer during 2016–2017 and they accounted for by 28.1% of all cancer diagnosed in women^2^. Breast cancer is a heterogenic disease with several subtypes and mostly are related to life style including alcohol and cigarettes consumption, overweigh, resistance to insulin, and to reproductive background including age at first gestation, early menarche, late menopause, hormonal therapy, null parity and aging^3^. Only 10% of all cases might be related to genetic inheritance and mutations on BRCA1 and BRCA2 genes^4^.

The search for a link between viruses and cancerous cells is not new and currently several types of human cancer are related to viral infections. Hepatocellular carcinoma, for instance, is related to Hepatitis B virus (HBV) and Hepatitis C virus (HCV) infection; human papillomavirus (HPV) genotypes 11 and 16 are found on cervical carcinoma and genital warts; the Epstein-Barr virus (EBV) is associated with Burkitt lymphoma and nasopharyngeal carcinoma; human T lymphotropic virus 1 (HTLV-1) causes T lymphocyte leukemia and human herpesvirus 8 virus (HHV-8)  is implicated with Kaposi sarcoma^5^. In general, it is estimated that 16% of all neoplastic diseases might be of viral origin^6^. However, although fragments of viral genomes have already been found in healthy and cancerous breast tissue, up to now no viral agent has been unequivocally linked to this type of cancer^7,8^.

New data on the link between a viral agent and breast cancer in women was recently published and indicated that an animal virus, the bovine leukemia virus (BLV), might be possibly associated with the etiopathogenesis of breast cancer^9^. BLV is a deltaretrovirus that causes a neglected, silent lifelong infection commonly found in dairy cattle, named enzootic bovine leukosis (EBL)^10^ which is characterized mainly by polyclonal expansion of CD5^+^ B lymphocytes^11^. Disease progression to B cell lymphoma is related to low expression of Tumor Necrosis Factor alpha (TNF-α), due to polymorphism in the promoter region^12^, and the animal’s Bola genotype^13^. The infection is considered eradicated in several parts of the world^14^ but is widely spread in North^15,16^ and most South American countries^17^ including Brazil. As most retroviruses, transmission requires transferring of cells from infected to non-infected individuals^18^ and, as such, blood and milk from infected animals are the main sources of the virus^19^.

Early studies indicated that BLV was non-infectious to humans^20^; however, by using sensitive serological and molecular approaches that theory is now refuted. At least one study indicated that human beings have antibodies to BLV capsid protein^21^ and, most importantly, several studies indicated that BLV DNA might be found in breast cancerous tissue at a higher rate than in healthy mammary tissue^9,22–25^. Nonetheless, to further incite this polemic issue, controversial data has been published indicating the absence of BLV inserts in DNA sequences from whole genome of breast tumors and normal breast tissue adjacent to the tumor retrieved from the NCBI database^26^ and in breast cancer tissue obtained from Chinese women^27^. The conflicting results could be related to the methodology applied, the genetic background of the population evaluated and eating habits such as high consumption of beef and milk.

Breast cancer is a public health issue in Brazil and BLV is widespread in Brazilian dairy cattle; in addition, consumption of meat and dairy product is higher in South Brazil compared to the other regions of the country. Thus, aiming to contribute to the discussion about breast cancer epidemiology and pathogenesis we carried out a study to find whether BLV DNA could be found associated with mammary tissue obtained from healthy breast tissue and from breast tissue with different carcinoma grade and if anti-BLV antibodies were present in healthy blood donors.

## Material and Methods

### Specimen selection

Formalin fixed paraffin embedded (FFPE) mammary tissue samples were obtained from the archives of the Instituto de Patologia de Passo Fundo, Rio Grande do Sul. The specimens were surgically removed during 2015 to 2017 and were from healthy women submitted to breast reduction surgery (n = 72) or women diagnosed with invasive breast carcinoma (n = 72), no special type, submitted to mastectomy or breast segmentectomy.

The pathological data contained the data and surgery type, patient age and microscopic diagnosis of the specimen. Samples were selected regardless the age, ethnicity or municipality origin of the subjects. Patients gave informed consent that unused tissue samples could be used at the discretion of the Institute. The study was approved by the Committee on Research Ethics (protocol # 2.247.462) of the Universidade de Passo Fundo, and the procedures used conformed to the tenets of the National Commission for Ethic on Research (CONEP) of the Brazilian Ministry of Health.

Immediately after surgical removal, breast tissue was fixed with 10% buffered formalin for 24–36 h and then embedded in paraffin blocks, sectioned (5 µm thick) and dried at −70 °C for 30 min and then used to make glass microscopic slides for hematoxylin eosin and immunostaining.One of the slides was used as a guide to select the areas of the paraffin block to be removed for DNA extraction. We removed areas containing higher amounts of mammary epithelial tissue (duct and lobules) using individual needles (1.6 mm diameter). The samples were then deparaffinized using standardized xylene, ethanol and water baths.

### Immunohistochemistry staining

For immunohistochemistry, dried sections were deparaffinized in xylene and dehydrated through graded alcohols to water. Antigen retrieval was achieved by boiling the sections in citrate buffer (pH 6.0); endogenous peroxidase activity was blocked by incubating the sections with peroxidase-blocking reagent. The slides were then incubated overnight (4 °C) with the following antibodies, all of them diluted 1:100: Ki-67 (Cell Marque, clone SP6), estrogen receptor (Cell Marque, clone SP1) progesterone receptor (Cell Marque, clone SP2), and HER2 (Cell Marque, clone SP3). Positive controls for all antibodies were included on all the slides. Immunostaining was processed using the chromogen diaminobenzidine (DAB) and all the slides were counterstained with Harris hematoxylin. The IHC analysis was read by two pathologists participating in this study, using a Zeiss Axio Scope A1 optical microscope. The interpretation of the immunohistochemistry followed the standards recommended by the American College of Pathology^28,29^.

### DNA extraction and Polymerase Chain Reaction

The DNA from the deparaffinized tissue samples was extracted using a commercial kit (RecoverAll™ Total Nucleic Acid Isolation Kit for FFPE - Ambion, Austin, TX, USA). Paraffinized lymph nodes from a calf knowingly negative and a cow knowingly positive to BLV, as assessed by Agar Gel Immunodiffusion (AGID), Indirect ELISA and PCR (27), was also used for DNA extraction and used as controls in the PCR reaction. In addition, a human DNA previously tested positive to BLV by PCR, kindly provided by Dr. Maria Fernandez Guttierez from the Pontificia Universidad Javeriana, Bogotá, Colombia^22^ was also used as positive control. DNA yield and quality was analyzed by spectrophotometry (NanoDrop, Thermo Scientific, USA).

For PCR, DNA quality was evaluated firstly by amplifying the human housekeeping gene glyceraldehyde-3-phosphate dehydrogenase (GAPDH) as reported previously^9^. In brief, 50 ng of DNA was used in a standard PCR reaction mix containing MgCl_2_ (2.0 mM/L) dNPTs (0.2 mM/L), Taq polimerase (0.025 U/μl) and forward (5′ GAGTCAACGGATTTGGTCGT-3′) and reverse (5′ TTGATTTTGGAGGGATCTCG-3′) primers (0.2 μM/L each) in a final volume of 25 µl at the following conditions: a 5 minutes initial denaturation cycle at 95 °C followed by 30 cycles, 30 second each, of DNA denaturing (95 °C), primer annealing (50 °C) and extension (72 °C), and then a final 10 minutes cycle of extension at 72 °C. The amplified fragments were analyzed by agar gel electrophoresis (2%) and stained using GelReady®.

All DNA samples that were amplifiable by the GAPDH PCR were then used in the nested PCR assay to detect BLV DNA according to the protocol published previously^9^ that was set to exclude the possibility of amplifying exogenous or endogenous retrovirus sequences. Again, 50 ng of DNA was mixed to a standard PCR mix equal to indicated above, but containing primers forward (5′-CTTCGGGATCCATTACCTGA-3′) and reverse (5′-GCTCGAAGGGGGAAAGTGAA-3′), at the same cycling conditions except for annealing temperature that in this case was set to 50 °C. Then, for the nested PCR (nPCR) assay, 5 µl from the previous PCR was transferred to a new micro tube containing forward (5′-ATGTCACCATCGATGCCTGG-3′) and reverse (5′-CATCGGCGGTCCAGTTGATA-3′) primers containing PCR reagents as indicated above. Cycling condition were the same except for the annealing temperature that was set to 56 °C. After nPCR the samples were analyzed by electrophoresis in 2% agar gel and stained with GelReady®. Control samples (bovine DNA from BLV positive and negative animals) and BLV positive human DNA^22^, and non-template samples were used throughout the assays to assure trustful results. Two DNA samples extracted from breast cancer tissue, which were positive to BLV by nPCR, were further evaluated by PCR containing primers targeting the capsid region of the BLV GAG gene^30^. The mixture content was then analyzed by agar gel electrophoresis and the resulting DNA fragments were excised from the gel and purified using a commercial kit aiming nucleotide sequencing.

### Blood serum samples and BLV blocking ELISA

One thousand five hundred human serum samples from a blood bank center were randomly selected and evaluated in this study. All samples were from healthy individual as described previously^31^. The presence of anti-BLV antibodies in serum samples was evaluated using a commercial anti-gp51 blocking ELISA kit (Ingezim BLV Compac 2.0, Spain). In brief, pools of 10 samples (20 µl each) were diluted (1:1) with the sample dilution buffer, added to the ELISA plate wells and incubated for 1 h at 37 °C. Then, the wells were washed and the peroxidase-labelled anti-gp51 monoclonal antibody was added, incubated and processed as recommended by the manufacturer. Negative and positive controls were provided in the kit. We made additional pools of positive and negative controls using 20 µl of serum from each sample as follows: a) a positive control consisting of 9 human serum that were negative when tested individually mixed to 1 bovine serum that was strongly positive; b) a second positive control consisting of the 9 human serum indicated above mixed to 1 bovine serum that was weakly positive; and c) a negative control made with the same 9 human blood samples mixed to a negative bovine serum sample. Samples from pools with a positive reaction were then evaluated individually to identify the positive samples. Negative and positive cut off values were calculated as indicated by the manufacturer.

### Statistical analysis

All statistical analyzes were performed using the software R 3.5.0 (https://www.r-project.org/). The relationship between the presence of BLV DNA and breast cancer was analyzed by T and Chi-square tests. Odds ratios (OR) were obtained and set with 95% confidence interval (CI). P values < 0.05 indicated statistically significant differences. Logistic regression models were used to estimate multivariate odds ratios for age and adjusted with 95% confidence interval.

## Results

**Figure 1:**
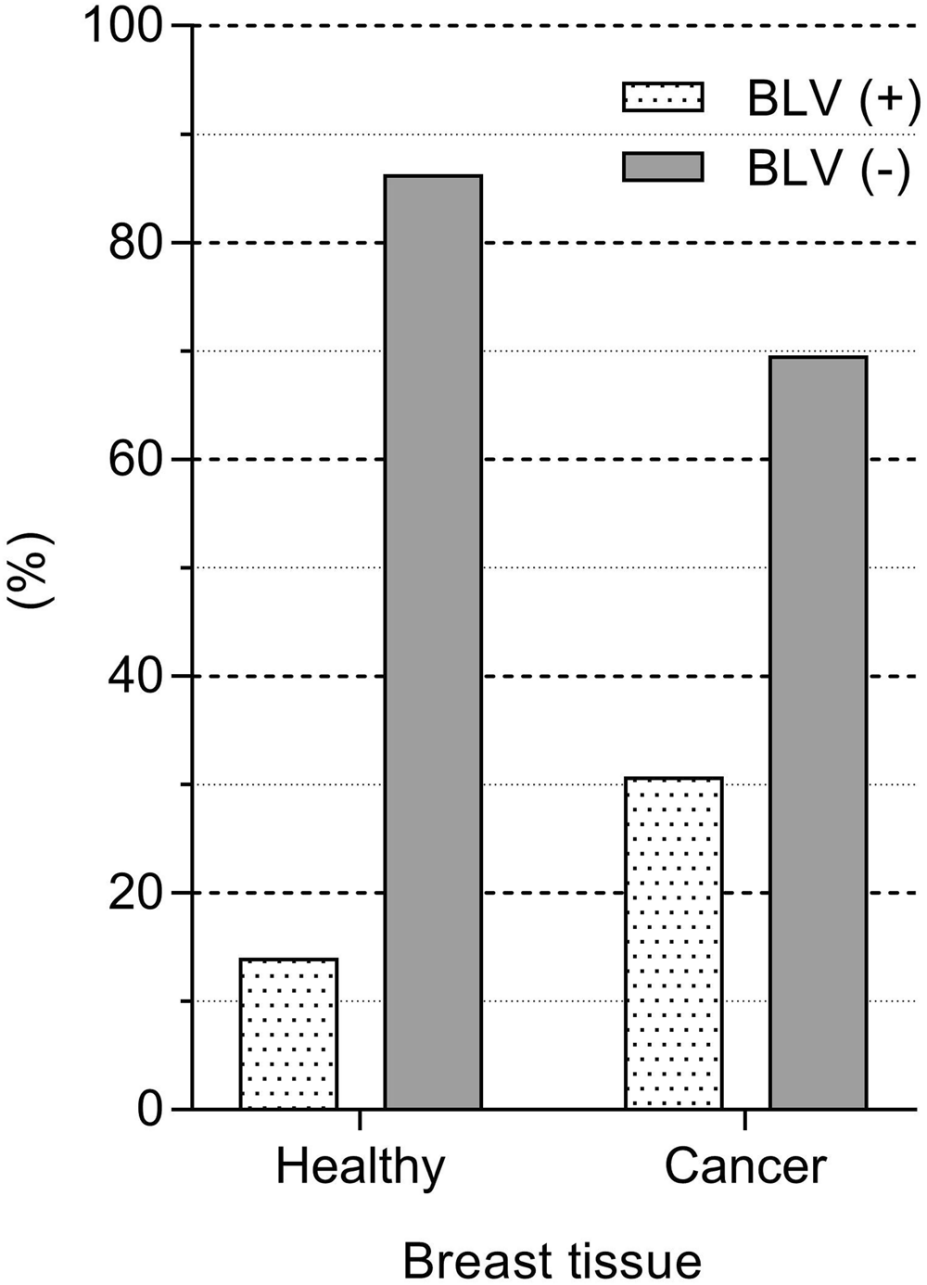
Bovine leukemia virus DNA in breast tissue. DNA from tissue samples without (n = 72) and with histological alterations (n = 72) were analyzed by nested PCR targeting BLV *Tax* gene. The data represent the percentage of samples with or without BLV DNA in each group (*Odd ratio* = 2.7).

**Figure 2:**
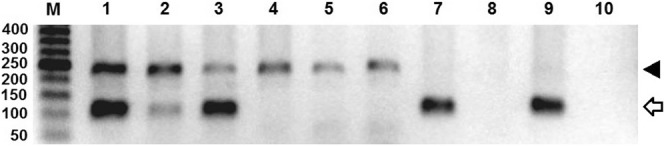
DNA fragments amplified from breast tissue samples. DNA quality was firstly analyzed by PCR targeting the GAPDH gene (arrow head, 237 bp) and then submitted to nested PCR targeting BLV *Tax* gene (open arrow, 113 bp). The gel was loaded using 5 ul of the PCR mix for the GAPDH gene and 5 ul from the nPCR reaction (Lanes 1 to 6). Lanes 1 to 3 depict the results of PCR amplification using DNA from breast cancerous tissue and lanes 4 to 6 represent the result of PCR amplification using DNA from healthy breast tissue. Lanes 7 and 8 contain the fragment amplified from human DNA previously found positive and negative to the presence of BLV DNA, respectively, as indicated in material and methods. Lanes 9 and 10 contain fragments amplified from bovine DNA knowingly positive and negative to BLV. The size of the molecular markers (M) is indicated on the left. The original full-length gel is presented in the Supplementary Information Fig. 1.

Overall, patients with breast cancer were older (median 52.5 years) than those without (median 38.0 years) and the median age of patient with or without BLV DNA was also slightly different, but not significant (Table 1). The relationship between the presence of BLV DNA and breast cancer was analyzed by T and Chi-square tests. BLV DNA was found in 30.5% (22/72) of the samples from breast cancer patient and on 13.9% (10/72) of samples from patients without neoplastic alterations on breast tissue (Fig. 1). These findings indicated a strong correlation between the presence of BLV DNA and breast cancer (*odds ratio* = 2.73; confidence interval = 1,18; p = 0.027). Detection of BLV *Tax* gene was carried out by nPCR on samples with a positive result on the GAPDH PCR (Fig. 2). Human DNA previously found positive to BLV and DNA from a cow knowingly positive to BLV were used as positive control. Two DNA fragment amplified from breast cancer tissue were sequenced and had 99% homology with the viral capsid protein gene (data not shown).

**Table 1:** Mean age of patients with or without cancer and with or without the presence of BLV DNA on breast tissue.

Samples	n	Age of the patient	
		Mean (SD)	Median
Breast cancer samples	72	52.4 (13.7)	52.5
Healthy breast samples	72	39.4 (14.6)	38.0
BLV (+) samples	32	50.2 (15.8)	52.5
BLV (−) samples	112	44.7 (15.3)	45.5
All samples	144	45.9 (15.6)	47.0

SD – standard deviation.

Other cellular markers that could be related to the presence of BLV DNA were investigated. The receptor for estrogen (ER) and progesterone (PR) were found in 91.0% of the breast cancer samples containing BLV and 88.0% of the samples from cancerous specimens without BLV and the presence of the Her-2 oncoprotein was similar in tumors with or without BLV DNA (Table 2).

**Table 2:** Overall characteristics of cancerous samples (n = 72) with and without BLV DNA. The results represent the percentage within each group.

Tumor Characteristics	Cancerous samples	
	BLV+ (n = 22)	BLV− (n = 50)
Tumoral grade 1	0.0%	14.0%
Tumoral grade 2	45.5%	48.0%
Tumoral grade 3	54.5%	38.0%
Lymph node (+)	72.7%	86.0%
Lymph node (−)	27.3%	14.0%
HR+	91.0%	88.0%
HR−	9.0%	12.0%
HER2+	13.6%	12.0%
HER2−	86.4%	88.0%
pT1c + pT2	91.0%	84.0%
pT1ab + pT3	9.0%	16.0%
Ki67 0–14	22.7%	32.0%
Ki67 14–30	18.1%	16.0%
Ki67 > 30	59.2%	52.0%

HR = hormonal receptor; HER2 = human epidermal growth factor receptor 2.

There was no correlation between the presence of BLV DNA and cancer metastasis. Metastasis in the sentinel lymph nodes were found in 72.7% of samples with and in 86.0% of samples without BLV DNA (Table 2). The size of the neoplastic tissue was not altered in BLV positive samples. In both groups 80.0% of the samples had 1 to 5 cm (pT1c and pT2). Most tumors were slightly or moderately differentiated (grade 2 and 3) either in BLV positive (100.0%) and negative (86.0%) samples.

The blocking ELISA is meant to evaluate the presence of antibodies to the viral main envelope glycoprotein (gp51) and to be used with bovine serum. However, as a blocking assay, the detecting antibody is an anti-BLVgp51 peroxidase-labelled monoclonal antibody that will bind to its cognate epitope when not blocked by a primary antibody. Thus, the specificity of the reaction is not species-specific. We evaluated the kit with bovine serum that were positive or negative by the traditional agar gel immunodiffusion (AGID) assay and then selected samples that were strongly and moderately positive, and negative in the ELISA, and used those samples to make the positive and negative pools. In this assay, the mean optical density (OD) reading of the negative and positive samples provided with the kit, measured in duplicates, were 1.614 and 0.116, respectively; based on these values, and using the formula provided in the instructions, samples with an OD ≥ 1.015 were considered negative and samples with an OD ≤ 0.865 were considered positive. Our controls consisting of negative human serum samples containing a bovine positive sample (pool “a”) had an OD of 0.059; pool “b” had an OD of 0.179 and pool “c” had an OD of 1.961 indicating that the assay was able to detect a single positive sample within 9 negative. Amongst 150 pools of 10 serum samples each (1500 samples) from healthy individuals we found only 2 positive pools; when assayed individually, 2 samples (OD 0.623 and 0.674) blocked binding of the anti-gp51 peroxidase-labelled monoclonal antibody and were thus considered positive to the presence of anti-BLV antibodies.

## Discussion

Breast cancer is a multifactorial disease that results from the interaction of several predisposing factors and represents a major burden to public health. The continuous search for a link between breast cancer and a viral agent indicated the possible involvement of BLV in driving cells to proliferate uncontrolled. BLV DNA was found in healthy mammary tissue, but also, and most frequently, in nonmalignant, premalignant, and malignant mammary tissue^9,22–25^. Also, antibodies to BLV capsid protein (p24) were detected in human blood^21^. More recently, BLV DNA was found in non-small cell lung cancer^32^. In contrast, in two independent studies, researchers could not find even traces of BLV DNA in mammary tissue with or without carcinoma^26,27^. In our study, however, BLV DNA was strongly correlated with breast cancer (*odds ratio* = 2,73). The conflicting data obtained from these studies might be related to the genetic background of the population evaluated or even to the methods applied in searching for a viral genome^9^. Nonetheless, when other classical risk factor for breast cancer are evaluated, we found that the link between BLV and cancer is higher than factors related to life style, hormone replacement therapy, and reproductive background, which have OR/RR values of 1,2 and 2,0, respectively. The association between BLV and cancer, in this case, is supplanted only by age, ionizing radiation and genetic factors^4^. In our study, individuals with breast cancer and BLV + individuals were older but when we adjusted the relative risk (odds ratio) for age we found that the difference was not significant and this might be accounted for by the relatively low number of samples (n = 72) evaluated in each group.

We found BLV DNA in 13.9% of mammary tissue with no histological sign of malignancy such as flat atypical epithelial, atypical intraductal hyperplasia and *in situ* carcinoma. This finding, however, was expected, in that the triggering factors leading to breast carcinogenesis have a long latency period up to clinical detection^33^. Furthermore, BLV is a deltaretrovirus intimately related to HTLV-1and both might remain at the cell nuclei but non-integrated to the genome during the asymptomatic and premalignant stages of leukemia/lymphoma^34,35^. During these stages the viral *Tax* protein inhibits DNA repair pathway mediated by the nucleotide excision repair system^36^. Thus, during the life span of the infected individual, DNA repair is inconsistent leading to genomic instability and neoplastic transformation^35–37^.

Finding a viral link with breast cancer tissues raises the question regarding the role of viruses in carcinogenesis. Different viruses have already been found in neoplastic mammary tissue^7,38^ and the presence of BLV DNA in our study could be a fortuitous finding; this idea has recently been refuted in that BLV DNA was found in breast tissue from patient prior to disease development^24^. Extracting DNA from areas with a high proliferation index and the possibility of cross-contamination could also contribute to ambiguous results. In our study we used a hematoxylin-eosin stained slide to guide us in selecting non-neoplastic (non-mitogenic) tissue from the paraffin block to extract DNA. We did so based on previous data which indicated that in 69.0% of breast cancer tissue the viral genome was found in tumoral and non-tumoral areas^9^. In addition, all procedures were performed under strict conditions to prevent any possibility of cross-contamination.

The mechanisms by which BLV reaches human beings have not been studied but it is likely related to the consumption of product derived from infected animals. The consumption of beef, milk and related product are similar in Brazil and Colombia^39^ and this could explain the similarity between the percentile of BLV DNA found in breast cancer patient in our study (30,5%) and those from Colombia (35,8%). In US and Australia, the amount of daily calories obtained from beef and dairy products is much higher^24,39^ and the percentage of BLV DNA associated to breast cancer was 59.0% and 80.0%, respectively.

The correlation between BLV and the molecular subtypes of carcinoma was also investigated. We had a limited number of patients positive and negative for the HER2 oncoprotein (HER2 subtype), therefore this correlation was not significant. We are aware that this type of cancer is less prevalent, has different triggering factors^40^, and a virus would have no major role on carcinogenesis when compared to cancer positive to hormonal receptors and normal expression of HER2 that were the subtypes most prevalent in our study. Other secondary factors that were correlated were tumor size, tumoral grade, KI67 index and the present of metastatic tissue on sentinel lymph nodes^41,42^. The possibility of metastatic tissue on sentinel lymph node is higher as the tumor size increases^41^; most of our patients had tumor ranging from 1 to 5 cm (pT1c and pT2) and a high number of patients with tumoral grade 2 and 3, highly mitotic and high KI67, which also contribute to metastatic draining lymph nodes^41,42^. Taken together, all tumor characteristics indicate a bad prognostic but no correlation with the presence of BLV DNA, most likely because of the limited number of samples evaluated.

Similarly to the detection of BLV DNA, the presence of antibodies to BLV in human serum has been a matter of dispute. In a previous study a high percentile of North American individuals had antibodies to the BLV p24 capsid protein^21^ contrasting with recent reports from a Chinese study that failed to demonstrate reactive antibodies to BLV in women with or without breast cancer^27^. The competitive ELISA we used is highly sensitive and not species-specific and thus was used to evaluate whether humans had antibodies directed to the viral glycoprotein. In our study only 2 out of 1500 samples (0.1%) had anti-BLV gp51 antibodies contrasting to the higher number of healthy mammary tissue containing BLV DNA; however, we are aware that the serological study might have a few shortcomings: first, gp51 is an envelope glycoprotein and as such is subject to variation within different viral subspecies^17^. Thus, the epitope targeted by the detecting monoclonal antibody could not be present on BLV subspecies that circulates in the region studied and, as such, infected individuals (bovine or human) might not have antibodies to that specific epitope and would be considered not infected in this assay. Second, different species might recognize distinctive epitopes on the same antigen; thus the suite of epitopes recognized by humans on a bovine virus are potentially different from those recognized by its natural host, and could explain the absence of antibodies to the epitope targeted by the monoclonal antibody in human serum. Furthermore, even amongst the natural host there might be individual that would render negative in one assay (e.g. blocking ELISA) but positive in an indirect ELISA using a different target antigen, such the viral capsid protein. Indeed, using an *in house* indirect ELISA^30^ based on a recombinant BLV capsid protein (p24r; sensitivity of 98.5% and specificity of 100.0%) and good correlation with the competing ELISA used here (κ = 0,68), we found bovine samples negative to gp51 by the blocking ELISA but positive to the p24r, and positive to the AGID and PCR assays, which indicates that antigenic variability might affect the outcome of the assay.

Taken together, our study provides further evidence on the involvement of BLV on breast cancer. Prospective studies with a larger number of samples might be required to strengthen our current finding and to incriminate (or not) BLV as a zoonotic agent with a major impact on public health. Then, the presence of BLV DNA in mammary tissue might become an important marker for breast cancer.

## Supplementary information

Supplementary information file

## Data Availability

Authors declare that materials, data and associated protocols used in this work are available upon request.
