# Supplementary material for: Bovine leukemia virus DNA associated with breast cancer in women from South Brazil
Source: Sci Rep. 2019 Feb 27;9:2949. doi: 10.1038/s41598-019-39834-7 (PMC6393560; doi:10.1038/s41598-019-39834-7)

Bovine leukemia virus DNA associated with breast cancer in women from South Brazil

Daniela Schwingel, Ana P. Andreolla, Luana M. S. Erpen, Rafael Frandoloso, Luiz C. Kreutz*

Running title: Bovine leukemia virus linked to breast cancer

Universidade de Passo Fundo, Laboratório de Microbiologia e Imunologia Avançada, Prédio G3. Campus I, Bairro São José, BR 285, Km 292. 99052-900 Passo Fundo, RS, Brazil. * Corresponding author (lckreutz@upf.br).

Supplementary figure S1. Original gel electrophoresis picture depicting GAPDH and BLV DNA fragments obtained after PCR assay on mammary tissue (Figure 2 on the article). In the article we show a clipping of this figure in which we removed the DNA ladder marker at the right side of the picture and the empty lane prior to the marker.

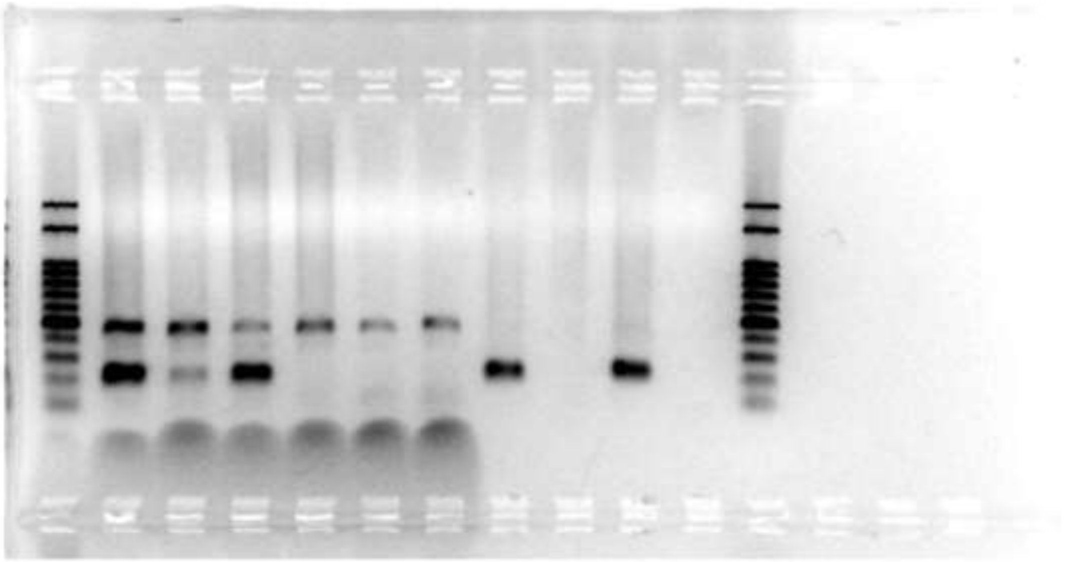

Supplement: Supplementary file 1 — Supplementary information file [file 41598_2019_39834_MOESM1_ESM.pdf]
